# Exposure to L-cycloserine incurs survival costs and behavioral alterations in *Aedes aegypti* females

**DOI:** 10.1186/1756-3305-7-373

**Published:** 2014-08-16

**Authors:** Virginia Belloni, Patricia Y Scaraffia

**Affiliations:** Department of Tropical Medicine, Vector-Borne Infectious Disease Research Center, School of Public Health and Tropical Medicine, Tulane University, 1430 Tulane Ave., SL-17, New Orleans, LA 70112 USA

**Keywords:** Mosquito behavior, Motor activity, Wing fanning, Knockdown response, Mortality, Amino acids, Mosquito metabolism, Survival

## Abstract

**Background:**

It was previously demonstrated that alanine aminotransferase (ALAT, EC 2.6.1.2) participates in maintaining the alanine-proline cycle between flight muscles and fat body during *Aedes aegypti* flight. ALAT is also actively involved in the metabolism of ammonia in *A. aegypti*. Here, we investigated the survival and behavioral costs of ALAT inhibition in *A. aegypti* females to better understand the role of ALAT in blood-fed mosquitoes.

**Methods:**

We analyzed how *A. aegypti* female mosquitoes respond to blood meals supplemented with 0, 2.5, 5 and 10 mM L-cycloserine, a well-known inhibitor of ALAT in animals. Mosquitoes were also exposed to blood meals supplemented with L-cycloserine and different concentrations of glucose (0, 10 and 100 mM). Additionally, the effects of ALAT inhibitor and glucose in mosquitoes starved for 24 or 48 h were investigated. Survival and behavioral phenotypes were analyzed during a time course (1, 2, 4, 6, 12, 24, 48 and 72 h after feeding).

**Results:**

L-cycloserine at 10 mM resulted in high mortality relative to control, with an acute effect during the first 6 h after treatment. A significant decrease in the number of active mosquitoes coinciding with an increase in futile wing fanning during the first 24 h was observed at all inhibitor concentrations. A high occurrence of knockdown phenotype was also recorded at this time for both 5 and 10 mM L-cycloserine. The supplementation of glucose in the blood meal amplified the effects of the ALAT inhibitor. In particular, we observed a higher mortality rate concomitant with an increase in the knockdown phenotype. Starvation prior to blood feeding also increased the effects of L-cycloserine with a rapid increase in mortality.

**Conclusions:**

Our results provide evidence that exposure of high doses of L-cycloserine during *A. aegypti* blood feeding affects mosquito survival and motor activity, suggesting an interference with carbohydrate and ammonia metabolism in a time-dependent manner.

**Electronic supplementary material:**

The online version of this article (doi:10.1186/1756-3305-7-373) contains supplementary material, which is available to authorized users.

## Background

Alanine aminotransferase (ALAT), also called glutamic-pyruvic transaminase (EC 2.6.1.2), is responsible for a bimolecular ping-pong reaction, where the α-amino group of alanine is transferred to α-ketoglutarate, leaving behind pyruvate and glutamate. This reversible reaction synthesizes alanine from pyruvate as well [1]. The enzyme contains the prosthetic group pyridoxal phosphate, which acts as a coenzyme in catalytic reactions.

In vertebrates, ALAT activity has been described in several different organisms such as fishes [2], amphibians [3], birds [4], and mammals [5–8]. ALAT is localized to both the cytosol and mitochondria [8, 9] and is widely distributed in several organs, with high levels in the liver and kidney [10–12]. In particular, ALAT is expressed in gluconeogenic tissues [8, 13]. In muscle, the enzyme synthesizes alanine from pyruvate, which is produced by glycolysis. Alanine is shuttled to the liver, where it is converted back to pyruvate and used for gluconeogenesis. The glucose produced is then delivered to the muscle to continue the cycle [13]. Moreover, ALAT plays an important role in the brain contributing to its energy supply [14].

In invertebrates, ALAT activity has been observed in crustacea [15], mollusks [16], and several insects, including locusts, tsetse flies, cockroaches, bees, moths [17], and mosquitoes [18]. In some insects, ALAT participates in the metabolism of proline during flight [18–21], and is involved in the metabolism of ammonia [22–24]. In addition, ALAT activity is present in the nervous system of some insects, such as bees [25, 26], waterbugs, cockroaches [27] and fruit flies [28], and provides energy to the nerve cells. For example, in the bee’s retina, glycogen accumulated in the glia is transferred to the neurons as pyruvate and alanine [25, 26].

Over the last few decades, a growing interest has developed in the effects of ALAT inhibition, in both vertebrates and invertebrates. Several potential inhibitors [2, 6, 11, 29] have been tested including β-chloro-L-alanine [30–32] and L-cycloserine (LCS), an L-isomer of 4-amino-3-isoxazolidinone [33]. LCS is one of the ALAT inhibitors most commonly used both *in vivo*[30, 34] and *in vitro*[7, 11, 16, 29, 30, 35].

In the present study, we evaluated the potential effects of ALAT inhibition in *A. aegypti,* a widely distributed species of mosquito and a primary vector of viral diseases such as dengue, yellow and chikungunya fever [36]. We investigated how *A. aegypti* females respond to different concentrations of LCS throughout a period of three days post blood meal, considering behavioral endpoints and mortality as indices of metabolic alterations. We also analyzed the effects of LCS and glucose in non-starved and starved *A. aegypti* females. The results presented in this manuscript demonstrate that high doses of LCS interfere with *A. aegypti* blood metabolism causing an impairment of important behavioral phenotypes and a high mortality.

## Methods

### Chemicals

L-cycloserine (LCS), D-glucose (Glc) and sucrose were purchased from Sigma-Aldrich (St. Louis, MO). Bovine blood was obtained from Pel-Freez Biologicals (Rogers, AR).

### Insects

*Aedes aegypti* (NIH Rockefeller strain, [37]) mosquitoes were reared at standard conditions as previously described [38]. Newly-eclosed mosquitoes were randomly assigned to different containers. Female mosquitoes were fed on 3% sucrose *ad libitum* until blood feeding or starved 24 or 48 h with access to water prior to a blood meal (BM). Mosquitoes were kept in a Caron 6015 Insect Growth Chamber, connected to a Caron CRSY 102 condensate recirculating System (Caron Products & Services, Inc., Marietta, OH) at 28°C, 75% relative humidity and a light: dark cycle of 16 h: 8 h until the end of the experiments.

### LCS treatments

Four-day-old female mosquitoes of the same size were allowed to feed for 15 min on blood meals (see below Treatment 1, 2 and 3) through an artificial blood feeder connected to a 37°C water bath [38]. Large groups of female mosquitoes were fed at the same time. After feeding, each female was carefully inspected and only fully engorged mosquitoes were individually transferred to 20 ml polyethylene vials (one female per vial). Each vial was covered with nylon mesh and secured with a rubber band. Mosquitoes were provided water *ad libitum* throughout the study period and maintained in an Insect Growth Chamber, as described above.

Mosquitoes underwent different experimental treatments:

- Treatment 1: BM supplemented with LCS (0, 2.5, 5, 10 mM). The experiment was replicated five times with five separate cohorts of mosquitoes, with a total sample size of 250 for each concentration.
- Treatment 2: BM supplemented with LCS (0, 10 mM) and Glc (0, 10, 100 mM). All the combinations of LCS and Glc were tested. The experiment was replicated three times with three separate cohorts of mosquitoes, with a total sample size of 75 for each concentration.
- Treatment 3: mosquitoes were starved for 24 or 48 h prior to the BM supplemented with LCS (0, 10 mM) and Glc (0, 100 mM). All the combinations of LCS and Glc were tested. The experiments were replicated three times with three separate cohorts of mosquitoes, with a total sample size of 75 for each concentration.

### Mortality and behavioral phenotypes

During a 15 sec window, mortality rate and behavioral observations were recorded at 1, 2, 4, 6, 12 (only in Treatment 1), 24, 48 and 72 h post blood meal (PBM).

Presence or absence of the following behavioral endpoints was individually scored:

- Active: mosquito shows normal behavior such as proper coordination and the ability to stand, walk, and fly.
- Wing fanning: mosquito shows a persistent wing fanning behavior in a futile attempt to fly. Wing fanning was previously described in other insects (reviewed in Haynes, 1988 [39]). It can be accompanied by loss of coordination.
- Knockdown: mosquito shows inability to stand, walk or fly, according to the ethological profile described in mosquitoes [40, 41].

In this manuscript the terms wing fanning and knockdown refer to impaired motor activity.

### Statistical analysis

Survival analysis was performed using Log-rank and Tarone-Ware tests. Bonferroni correction was applied when multiple comparisons were performed. The analysis was carried out using GraphPad Prism version 6.0 for Mac OS X (GraphPad Software, San Diego, CA). Behavior was categorized into three levels (active, wing fanning and knockdown). Generalized estimating equation (GEE) regression methods were used to examine the impact of treatment, and any interaction with time, on behavior. Behavioral data were analyzed using PROC GENMOD, SAS version 9.3 (SAS Institute Inc., Cary, NC) with a cumulative logit link and an independent correlation structure. A *p*-value less than 0.05 was considered significant. Data are presented as mean ± standard error (SE).

## Results

### LCS impairs motor activity and survival

To better understand the role of ALAT in blood-fed *A. aegypti* metabolism, we experimentally assessed the survival costs of LCS-dependent inhibition in *A. aegypti* females and analyzed their behavioral phenotypes during 3 days post treatment. This was done by comparing outcomes between mosquitoes that were fed a BM (control group) and mosquitoes fed BM’s with LCS at varying concentrations (Treatment 1).

**Figure 1:**
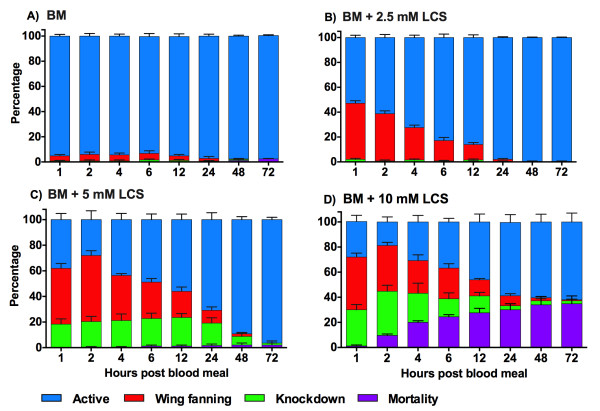
**Effect of LCS on the behavioral phenotypes and mortality in** ***A. aegypti*** **. A)** BM; **B)** BM + 2.5 mM LCS; **C)** BM + 5 mM LCS; **D)** BM + 10 mM LCS. BM: blood meal; LCS: L-cycloserine. Female mosquitoes were fed a blood meal supplemented with different concentrations of LCS (0, 2.5, 5 and 10 mM). Data are expressed as mean percentage ± SE.

As shown in Figure 1, only 5 (2% ± 1) of the individuals fed with blood alone (BM) died during the 72 h of observation, while mosquitoes exposed to 10 mM LCS (BM + 10 mM LCS) had a significantly higher mortality (35% ± 6) [Log-Rank, χ^2^_3_ = 259.2, *p* = 0.0001] than the other three groups (BM = 2% ± 1; BM + 2.5 mM LCS = 0.4% ± 0.4; BM + 5 mM LCS = 2% ± 1). Moreover, at 10 mM LCS most mortality occurred within the first hours PBM, reaching 24% by 6 h, while only 11% of the mosquitoes died between day 1 and day 3. No difference in mortality was observed among the other groups of this treatment [Log-Rank, χ^2^_2_ = 3.55, *p* = 0.17].

When we examined behavioral phenotypes (Figure 1; Additional file 1: Video S1) of all four groups, motor activity was impaired in a time-dependent manner by LCS administration [GEE, Time, χ^2^_1_ = 507.24, *p* = 0.0001], resulting in a greater occurrence of wing fanning and knockdown behavior relative to control. The LCS effect was dose-dependent, where the number of active mosquitoes decreased with the increase of LCS concentration accompanied by a higher probability of knockdown response [GEE, Dose, χ^2^_1_ = 273.54, *p* = 0.0001]. Wing fanning was observed at all LCS concentrations, while knockdown was almost absent at 2.5 mM LCS (Figure 1A-D; Additional file 2: Table S1). Impairment of motor activity occurred within the first hours PBM. However, a clear recovery was observed over time (Figure 1B-D).

### LCS and Glc increase the behavioral response and mortality

Since pyruvate required for transamination can be produced by the oxidation of Glc, we speculated that the effects of LCS on mosquitoes would be modified in the presence of Glc. To determinate whether Glc alters the effects of LCS on mosquitoes, we investigated the survival costs of the ALAT inhibitor in *A. aegypti* females after they were provided blood meals supplemented with LCS and Glc (Treatment 2). As with Treatment 1, we evaluated their behavioral responses during 72 h PBM.

**Figure 2:**
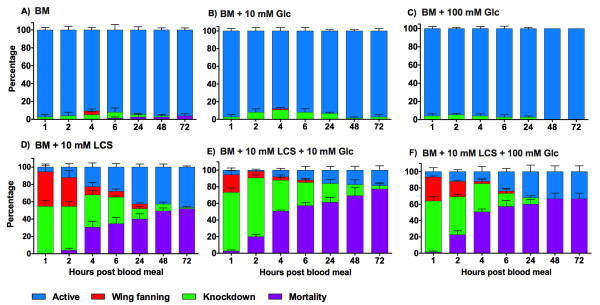
**Effect of LCS and Glc on the behavioral phenotypes and mortality in** ***A. aegypti*** **. A)** BM; **B)** BM + 10 mM Glc; **C)** BM + 100 mM Glc; **D)** BM + 10 mM LCS; **E)** BM + 10 mM LCS + 10 mM Glc; **F)** BM + 10 mM LCS + 100 mM Glc. BM: blood meal; LCS: L-cycloserine; Glc: glucose. Female mosquitoes were fed a blood meal supplemented with different concentrations of LCS (0, 10 mM) and Glc (0, 10, 100 mM). Data are expressed as mean percentage ± SE.

During the course of the experiment no mosquitoes died in the groups exposed to BM + 10 mM Glc or BM + 100 mM Glc when compared to BM (Figure 2). When the effect of 10 mM LCS was tested, mortality was significantly higher than in the BM control [Log-Rank, χ^2^_3_ = 89.76, *p* < 0.0001; BM: 4% ± 2; BM + 10 mM LCS: 52% ± 0; BM + 10 mM LCS + 10 mM Glc: 77% ± 7; BM + 10 mM LCS + 100 mM Glc: 67% ± 7] (Figure 2). In the presence of Glc, the effect of LCS increased [Log-Rank, χ^2^_2_ = 12.37, *p* = 0.0021; BM + 10 mM LCS; BM + 10 mM LCS + 10 mM Glc; BM + 10 mM LCS + 100 mM Glc], reducing the population by half at 4 h PBM. When BM + 10 mM LCS + 10 mM Glc and BM + 10 mM LCS + 100 mM Glc were compared, no dose-dependent effect was observed (Figure 2).

As shown in Figure 2A-C, no difference in the behavioral phenotypes was observed among the BM, BM +10 mM Glc and BM + 100 mM Glc groups, while LCS significantly affected mosquito activity in a time-dependent way [GEE, Time, χ^2^_1_ = 40.61, *p* = 0.0001]. When we compared the effect of the inhibitor under Glc supplementation (0, 10 and 100 mM), Glc decreased the probability of active behavior [GEE, Dose Glc: χ^2^_2_ = 34.39, *p* = 0.0001] due to an increase of knockdown response (Figure 2). However, the effect was independent from the Glc concentration (Additional file 3: Table S2), and over time BM + 10 mM LCS + 100 mM Glc group recovered, reaching a number of active mosquitoes similar to BM + 10 mM LCS group. A slight recovery was observed in BM + 10 mM LCS + 10 mM Glc group [GEE, Time*Dose Glc: χ^2^_2_ = 14.64, *p* = 0.0007; Figure 2D-F].

### Starvation prior to LCS-treatment severely increases the LCS effects

It is well known that starvation mobilizes the nutritional reserves in both vertebrates and invertebrates. To explore whether starvation impacts the phenotypes observed after LCS treatment, we tested the survival costs of the ALAT inhibitor on mosquitoes starved for 24 or 48 h (Treatment 3). We also examined their behavioral responses during 3 days post treatment.

**Figure 3:**
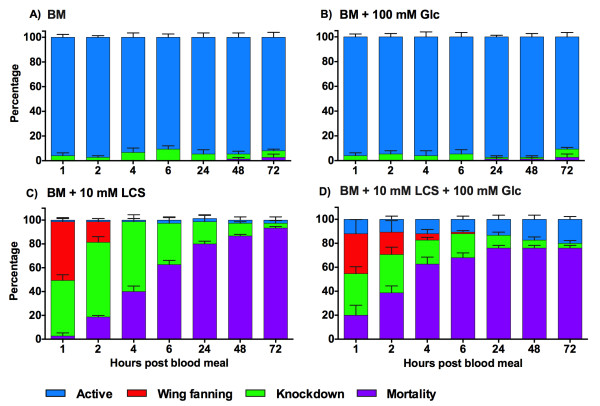
**Effect of LCS on the behavioral phenotypes and mortality in 24 h starved** ***A. aegypti*** **. A)** BM; **B)** BM + 100 mM Glc; **C)** BM + 10 mM LCS; **D)** BM + 10 mM LCS + 100 mM Glc. BM: blood meal; LCS: L-cycloserine; Glc: glucose. Female mosquitoes were starved for 24 h prior to the administration of a blood meal supplemented with LCS (0, 10 mM) and Glc (0, 100 mM). Data are expressed as mean percentage ± SE.

Starvation for 24 h significantly affected mosquito survival in the presence of LCS (BM + 10 mM LCS: 93% ± 1; BM + 10 mM LCS + 100 mM Glc: 76% ± 2) relative to the control groups (BM: 3% ± 3; BM + 100 mM Glc: 3% ± 3) [Tarone-Ware, χ^2^_3_ = 219.1, *p* < 0.0001] (Figure 3). Although a similar trend was observed in LCS-treated groups, the rate of mortality increased faster in the presence of Glc during the first 24 h, where 50% mortality was reached by 4 h PBM. The effect was stronger later in the absence of Glc with a higher total mortality at 72 h PBM (Figure 3).

As shown in Figure 3 and Additional file 4: Table S3, the high mortality rate in LCS groups was associated with a reduced number of active mosquitoes and a greater knockdown response, compared to control groups (BM and BM + 100 mM Glc). Wing fanning was also observed shortly after feeding in LCS groups. Time was not a significant factor [GEE, Time: χ^2^_1_ = 0.01, *p* = 0.9] with few active mosquitoes in the LCS groups. However, Glc supplementation resulted in a higher probability of active behavior and lower knockdown response associated with a greater percentage of survival [GEE, Treatment Glc, χ^2^_1_ = 13.73, *p* = 0.0002; Time*Treatment Glc, χ^2^_1_ = 5.04, *p* = 0.0248; Figure 3C-D].

**Figure 4:**
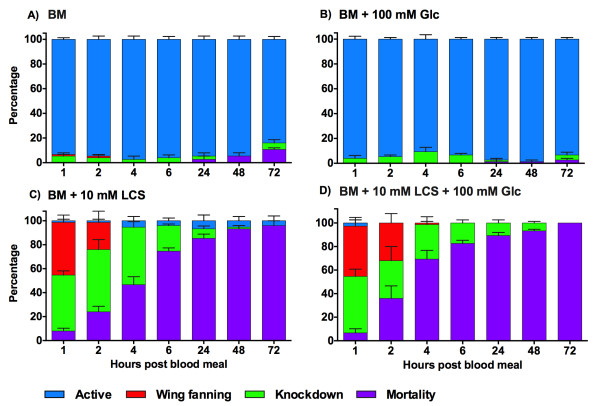
**Effect of LCS on the behavioral phenotypes and mortality in 48 h starved** ***A. aegypti*** **. A)** BM; **B)** BM + 100 mM Glc; **C)** BM + 10 mM LCS; **D)** BM + 10 mM LCS + 100 mM Glc. BM: blood meal; LCS: L-cycloserine; Glc: glucose. Female mosquitoes were starved for 48 h prior to the administration of a blood meal supplemented with LCS (0, 10 mM) and Glc (0, 100 mM). Data are expressed as mean percentage ± SE.

When mosquitoes were starved for 48 h prior to blood feeding (Figure 4), a similar response pattern to 24 h starvation was observed; LCS treatment resulted in significant mortality (BM + 10 mM LCS: 96% ± 4; BM + 10 mM LCS + 100 mM Glc: 100%) compared to the control groups (BM: 11% ± 1; BM + 100 mM Glc: 3% ± 1) [Log-Rank, χ^2^_3_ = 319.9, *p* < 0.0001], with a faster increase in the mortality rate in the first 24 h PBM in the presence of Glc (median survival = 2 h PBM).

The LCS-treated groups showed a low occurrence of active behavior and a high occurrence of knockdown response, which reached its highest level within the first hours PBM with respect to the groups not exposed to LCS (Figure 4 and Additional file 5: Table S4). Furthermore, the same mosquitoes showed wing fanning behavior only shortly after feeding (1–2 h) (Additional file 5: Table S4). When the effect of Glc was considered (BM + 10 mM LCS + 100 mM Glc), no recovery was observed. In absence of Glc (BM + 10 mM LCS), LCS-survived mosquitoes recovered slightly over time [GEE, Treatment Glc, χ^2^_1_ = 17.39, *p* < 0.0001; Time*Treatment Glc, χ^2^_1_ = 11.75, *p* = 0.0006; Figure 4C-D].

## Discussion

Alanine aminotransferase (ALAT) plays an important role in maintaining the alanine-proline cycle between flight muscles and fat body during *A. aegypti* flight [18]. Additionally, the ALAT enzyme responds efficiently when *A. aegypti* mosquitoes face an ammonia challenge [22–24]. Thus, any interference with ALAT activity could compromise the efficiency of the pathways involved in ammonia metabolism [22–24, 38, 42], resulting in deleterious effects on blood-fed *A. aegypti* females. In the study performed here, we treated *A. aegypti* mosquitoes with L-cycloserine (LCS), a well-known inhibitor of ALAT.

In animals, LCS is able to strongly or completely inhibit ALAT activity [7, 11, 16, 29, 30, 34]. When increasing concentrations of LCS (0, 2.5, 5 and 10 mM) in blood meals were tested in *A. aegypti* females (Treatment 1, Methods Section), only the highest dose affected female survival with an acute effect during the first hours post treatment. However, motor behavior was affected in all the LCS-groups following a time-dependent pattern with a clear recovery over time. All three concentrations of LCS showed a high occurrence of wing fanning, but only 5 and 10 mM LCS were associated with a significant number of mosquitoes exhibiting the knockdown behavior. Mortality was observed only at 10 mM LCS, suggesting a dose-dependent increase in the severity of the motor disruption with death as the ultimate impairment. Interestingly, the addition of glucose into blood meals (Treatment 2, Methods Section) or starvation (Treatment 3, Methods Section) increased the LCS effects on mosquitoes. Our data also indicate that behavioral endpoints are a useful tool to investigate the effect of enzymatic inhibitors able to interfere with mosquito metabolism. Motor activity impairment caused by the exposure to chemicals in natural conditions could result in a compromised seeking and biting behavior, and therefore affect disease transmission [43, 44].

In LCS-blood fed *A. aegypti* (Treatments with 10 mM LCS), the early peak observed in both mortality and impaired behavior suggests that LCS strongly interacts with ALAT soon after blood feeding. Early LCS response was also described in rats, where inhibition of ALAT activity rapidly reached a peak merely 30–60 minutes post LCS injection [34]. One hour LCS-perfusion also resulted in a fast decrease of ALAT in rats [45]; whereas in mice an intra-peritoneal injection of the inhibitor affected ALAT and induced a moderate impairment of motor performance three hours after administration [46]. The recovery observed in surviving LCS-blood fed mosquitoes might indicate that the inhibitor acts rapidly on tissues, but somehow it is then detoxified or removed from the system. The underlying mechanisms of mosquito recovery are unknown at the present. However, it is possible that the surviving mosquitoes use alternative pathways to deal with ALAT inhibition, as previously reported in blood-fed *A. aegypti* females when glutamine synthetase was silenced by specific inhibitors [22–24].

The phenotypes described here in *A. aegypti* mosquitoes treated with high doses of LCS could be associated with an alteration of ammonia metabolic pathways. In *A. aegypti*, inhibition of enzymes involved in fixation and assimilation phases of ammonia metabolism [22–24] resulted in a high mortality, preceded by the suppression of locomotor activity [24]. In mammals, an excess of ammonia in the brain affected the activity of neurotransmitters involved in the regulation of motor activity such as acetylcholine [47], glutamate and its product γ-aminobutyric acid [48–50]. High levels of ammonia in neuronal and other tissues have been associated with deleterious effects and death in several animal species [48, 51–53] owing to an increase in oxidative stress, energy deficiency, and alteration of neurotransmission systems in a concentration and time-dependent manner [48, 51, 54]. Recently, Beuster *et al.*, [30] found that ALAT inhibition with LCS correlates with several alterations in mammalian cancer cells, such as energy deficiency, increased respiration rates, and mitochondrial production of reactive oxygen species. Moreover, inhibition of ALAT by LCS was associated with a decrease in glucose uptake, and thus a suppression of the overall glucose metabolism in rodent cell lines [30]. In *A. aegypti* mosquitoes, sugar feeding plays an important role in the metabolism of amino acids and energy supply [55]. In Treatment 2, all the LCS-groups showed a similar time-dependent pattern associated with recovery over time, although lower in the presence of glucose. The severity of LCS effects after glucose supplementation observed here correlates closely with a disruption of glucose and amino acid metabolism in *A. aegypti* females. It could be interesting to explore whether any of these cellular effects caused by LCS in vertebrates can be also correlated to the behavioral alterations observed in mosquitoes treated with the higher doses of LCS.

In *A. aegypt*i, starvation strongly impacts energy supply and results in low flight potential [56]. Moreover, when *A. aegypti* are sugar-starved for 24 h prior to feeding on a protein meal, proline and alanine levels are significantly increased relative to the level of non-starved mosquitoes [57]. In rodents, 48 h starvation induces an increase in alanine production, while alanine release is decreased by LCS perfusion [58]. These findings could support how starvation can result in the greater mortality and behavioral alterations observed in *A. aegypti* mosquitoes exposed to ALAT inhibitor. The LCS administration induced a rapid increase in mortality in *A. aegypti* females starved for 24 h. The effect was amplified in females starved for 48 h. In addition, the supplementation of glucose resulted in a faster increase in the mortality, but only in the first 4 h post treatment. Finally, a slight recovery and reduced number of knockdown phenotype occurred in the presence of glucose in mosquito females starved for 24 h, while no recovery was observed in females starved for 48 h.

Despite high specificity of LCS for ALAT, it is known that LCS can interact with other transaminases in animals [11, 34, 46]. Therefore, we cannot exclude the possible side effects of the LCS treatment on additional targets in mosquitoes. To overcome this problem, we are currently investigating the effects of silencing ALAT in *A. aegypti* females through RNA interference.

## Conclusion

This study demonstrates that exposure to high LCS doses incurs survival costs and behavioral alterations in *A. aegypti* females. The LCS effects were amplified when mosquito blood meals were supplemented with glucose or when females were starved prior to blood feeding. Taken together, our data suggest that LCS interferes with carbohydrate and ammonia metabolism in a time-dependent manner in blood-fed *A. aegypti* females.

## Electronic supplementary material

Additional file 1: Video S1: Effect of LCS on wing fanning behavior in an *A. aegypti* female. Mosquito behavior was video recorded in an *A. aegypti* female exposed to a blood meal supplemented with LCS (10 mM). The observation was recorded at 4 h post blood meal. (MOV 4 MB)

Additional file 2: Table S1: Comparison of behavioral phenotypes in *A. aegypti* exposed to LCS (0, 2.5, 5 and 10 mM) (Treatment 1, Methods Section). Data are expressed as mean percentage (±SE) calculated from the surviving mosquitoes. (XLSX 40 KB)

Additional file 3: Table S2: Comparison of behavioral phenotypes in *A. aegypti* exposed to LCS (0, 10 mM) and Glc (0, 10, 100 mM) (Treatment 2, Methods Section). Data are expressed as mean percentage (±SE) calculated from the surviving mosquitoes. (XLSX 41 KB)

Additional file 4: Table S3: Comparison of behavioral phenotypes in *A. aegypti* starved for 24 h and exposed to LCS (0, 10 mM) and Glc (0, 100 mM) (Treatment 3, Methods Section). Data are expressed as mean percentage (±SE) calculated from the surviving mosquitoes. (XLSX 41 KB)

Additional file 5: Table S4: Comparison of behavioral phenotypes in *A. aegypti* starved for 48 h and exposed to LCS (0, 10 mM) and Glc (0, 100 mM) (Treatment 3, Methods Section). Data are expressed as mean percentage (±SE) calculated from the surviving mosquitoes. (XLSX 41 KB)

## Abbreviations

ALAT: Alanine aminotransferase
LCS: L-cycloserine
Glc: Glucose
BM: Blood meal
PBM: Post blood meal.
